# Integrated analysis of electrical stimulation effects on *Pseudomonas aeruginosa* PAO1 inoculated denitrifying community: targeted and untargeted metabolomic analysis of phenazine biosynthesis and quorum sensing

**DOI:** 10.3389/fmicb.2025.1621417

**Published:** 2025-06-13

**Authors:** Li Wu, Yong Liu, Jianping Deng, Shuanglin Gui, Hanbing Nie

**Affiliations:** ^1^Jiangxi Provincial Key Laboratory of Greenhouse Gas Accounting and Carbon Reduction, Institute of Energy Research, Jiangxi Academy of Sciences, Nanchang, China; ^2^Jiangxi Carbon Neutalization Research Center, Nanchang, China

**Keywords:** *Pseudomonas aeruginosa* PAO1, quorum sensing, phenazine derivative, microbial electrolysis cell, untargeted metabolomic analysis

## Abstract

This study investigates how 0.8 V applied voltage modulates phenazine biosynthesis, quorum sensing (QS), and microbial interactions in *Pseudomonas aeruginosa* PAO1-inoculated microbial electrolysis cell (MEC) reactors. Voltage stimulation significantly enhanced phenazine derivatives (PYO: 8.65-fold; 1-OH-PHZ: 14.98-fold) and QS signals (C4-HSL: 2.88-fold; 3-OXO-C12-HSL: 2.21-fold), correlating with upregulated biosynthetic genes (phzG: 14.8-fold; rhlI: 15.2-fold). Electrical stimulation amplified QS cross-regulation, reinforcing Las-mediated positive feedback on Rhl/PQS systems while attenuating Rhl’s inhibition of PQS. Untargeted metabolomic analysis demonstrated significant alterations in bacterial metabolic activity under electrical stimulation, identifying 140 differential metabolites. Among these, indole, a signaling molecule with QS-like functionality, exhibited the highest VIP score as an upregulated metabolite, and another indole derivative, brassicanal A, was also elevated. KEGG pathway enrichment analysis highlighted that these metabolites were primarily associated with amino acid metabolism and transport, while anthranilic acid and L-tryptophan—key metabolites linked to both indole-related pathways and phenazine biosynthesis—were also identified. Correlation analysis between differential metabolites with microbial communities confirmed that *Delftia* and *Burkholderiales* were strongly associated with phenazine biosynthesis and QS activity in *P. aeruginosa* PAO1. These findings highlight voltage as a key driver of metabolic rewiring and microbial niche partitioning, optimizing MEC reactor performance for wastewater treatment. This work provides foundational insights into electro-stimulated biofilm engineering through targeted QS and metabolic pathway regulation.

## 1 Introduction

*Pseudomonas aeruginosa*, a Gram-negative opportunistic pathogen, employs a sophisticated quorum sensing (QS) system to coordinate population-wide behaviors critical for virulence and environmental adaptation (Li et al., 2022; Shariati et al., 2024). In the medical field, researchers have attempted to suppress the survival capability of *Pseudomonas aeruginosa* by quenching quorum sensing to treat wound infections caused by this pathogen (Rather et al., 2022; Rodriguez-Urretavizcaya et al., 2024). However, *P. aeruginosa* has also emerged as a pivotal microbial agent in wastewater nitrogen removal due to its unique metabolic versatility and engineering adaptability (Nie et al., 2020). Regulating its quorum sensing through specific methods can enhance its competitive advantage in water treatment systems, thereby achieving high-efficiency nitrogen removal.

In *P. aeruginosa* PAO1, the well-characterized QS systems with defined regulatory mechanisms are primarily the N-acyl-homoserine lactone (AHL)-based QS systems (Las and Rhl) and the PQS (Pseudomonas Quinolone Signal) system (Schuster and Greenberg, 2006; Vadakkan et al., 2024). The Las system is composed of the LasR protein (acting as a signal receptor) and the LasI protein (acting as a signal emitter). The LasI protein is primarily responsible for synthesizing the signaling molecule N-3-oxododecanoyl-L-homoserine lactone (3-oxo-C12-HSL), which is then exported outside the cell membrane (de Oliveira Pereira et al., 2023). Free 3-oxo-C12-HSL molecules are detected by LasR proteins on neighboring cell membranes, forming a complex that activates the Las system and subsequently regulates the expression of downstream genes. This system mainly influences metabolic activities such as the synthesis of elastase, alkaline protease, proteolytic enzymes, toxin A, and the formation of pili and flagella. The Rhl system consists of the RhlR signal receptor protein and the RhlI signal synthase protein. The RhlI protein generates the signaling molecule N-butanoyl-L-homoserine lactone (C4-HSL) within the cell, which binds to RhlR to regulate downstream gene expression. This system primarily governs the synthesis of elastase and alkaline protease, and is also associated with the production of phenazine derivatives and rhamnolipids (Latifi et al., 1995; Pearson et al., 1997).The PQS system involves the operons *pqsABCDE*, *phnAB*, and *pqsH*, which form a genetic cluster controlling the synthesis of quorum-sensing PQS molecules. Upon activation, the PQS system regulates metabolic activities such as biofilm formation, phenazine derivative synthesis, and protease production in *Pseudomonas aeruginosa* (Gallagher et al., 2002; Rampioni et al., 2010; Winzer and Williams, 2001).

Phenazine derivatives are endogenous electron shuttles primarily secreted by *Pseudomonas* species, facilitating indirect extracellular electron transfer (IEET) (Franco et al., 2024; Saunders et al., 2020). In *P. aeruginosa* PAO1, two operons, *phzA1B1C1D1E1F1G1* and *phzA2B2C2D2E2F2G2*, independently control the synthesis of simple phenazine compounds such as phenazine-1-carboxamide (PCN) without mutual interference (Cui et al., 2016; Sun et al., 2019). Subsequently, PCN and phenazine-1-carboxylic acid (PCA) are modified by proteins encoded by the *phzS*, *phzM*, and *phzH* genes into derivatives such as 1-hydroxyphenazine (1-OH-PHZ) and pyocyanin (PYO) (Huang et al., 2020; Qiao et al., 2015, 2017). *P. aeruginosa* utilizes these phenazine derivatives for electrogenic respiration by anchoring to MFC anode electrodes and enhances biofilm formation (Price-Whelan et al., 2006; Ramos et al., 2010). Previous studuy have demonstrated that exogenous supplementation of 3-oxo-C12-HSL can enhance phenazine derivatives secretion, biomass production, and denitrification activity during the early stages of *P. aeruginosa* PAO1 inoculation, thereby improving its competitive advantage in water treatment processes (Nie et al., 2021). In another study, electrical stimulation of *P. aeruginosa* PAO1 achieved similar outcomes (Nie et al., 2022). However, the research failed to establish a direct connection between the electrical stimulation and quorum sensing regulation.

This study aims to investigate the phenazine derivatives, quorum sensing signal molecules, and related gene expression of *P. aeruginosa* PAO1 in previously operated MEC reactors under conditions with and without 0.8V applied voltage. Through integration with untargeted metabolomics analysis, the work further seeks to identify metabolites closely associated with QS in the communities of *P. aeruginosa* PAO1 inoculated under electrical stimulation. Therefore, elucidating the relationship between the electroactivity of *P. aeruginosa* PAO1 and QS is critical for optimizing its application in wastewater treatment.

## 2 Materials and methods

### 2.1 Samples

All samples in this experiment were derived from MEC reactors inoculated with *Pseudomonas aeruginosa* PAO1 operating for 72 days under conditions of 0.8 V applied voltage (corresponds to anode potential) and non-electrified control in previous studies (Nie et al., 2021). For targeted metabolomic analysis of phenazine synthesis and quorum sensing: 50 mL of bacterial culture from this stage was centrifuged at 8,000 rpm for 5 min. The supernatant was collected for detecting phenazine derivatives and quorum sensing signal molecule concentrations, while the pellet (bacterial cells) was used for RNA extraction followed by reverse transcription and real-time quantitative PCR (RT-qPCR) analysis. For untargeted metabolomic analysis: 50 mL of the same bacterial culture was centrifuged at 8,000 rpm for 5 min to remove the supernatant. The remaining cell pellet was freeze-dried for 24 h and stored at −80°C for subsequent detection.

### 2.2 Detection of phenazine derivatives

The concentrations of phenazine derivatives were determined using methods established in previous studies (Nie et al., 2021). For the extraction of 1-hydroxyphenazine (1-OH-PHZ) and pyocyanin (PYO), 5 mL of supernatant was transferred to a 10 mL centrifuge tube, mixed with 5 mL of chloroform, and vigorously vortexed for 5 min to fully dissolve 1-OH-PHZ and PYO into the chloroform phase. After centrifugation at 10,000 rpm for 5 min to separate the phases, the upper aqueous layer was discarded. A secondary extraction was then performed by adding 5 mL of pH 10 sodium hydroxide (NaOH) solution for 1-OH-PHZ and pH 3 hydrochloric acid (HCl) solution for PYO, following the same vortexing and centrifugation steps. The final aqueous layer was retained, and absorbance was measured at 520 nm to determine concentrations based on standard curves. For phenazine-1-carboxylic acid (PCA) extraction, only a single-step chloroform extraction was performed using the same protocol, but the lower chloroform phase was directly analyzed by measuring absorbance at 252 nm. Standards for PCA and 1-OH-PHZ were purchased from Macklin Biochemical (Shanghai, China), and the PYO standard was obtained from Cayman Chemical (Ann Arbor, United States).

### 2.3 Detection of AHL-mediated QS signal molecules

The supernatant samples were mixed with an equal volume of ethyl acetate acidified with 0.1% formic acid. Two sequential extractions were performed to ensure complete recovery of target compounds. The combined ethyl acetate extracts were concentrated to 2 mL using a rotary evaporator at 40°C, followed by further evaporation to 400 μL under a nitrogen stream at 40°C. The aqueous phase was analyzed for C4-HSL, C6-HSL, C8-HSL, C10-HSL, and 3-oxo-C12-HSL using liquid chromatography-mass spectrometry (LC-MS; Thermo Scientific UltiMate 3000 UHPLC-Q Exactive system, Waltham, MA, United States) (Liu et al., 2024). AHLs standard samples were purchased from Yuanye Biotechnology (Shanghai, China).

### 2.4 RNA extraction and RT- qPCR

The samples were aliquoted into 50 mL centrifuge tubes and subjected to centrifugation at 8,000 rpm for 5 min to concentrate bacterial biomass (Liu et al., 2019). Total RNA was isolated from the pelleted cells using the RNAprep Bacteria Kit (Aidlab Biotechnologies, China), followed by DNase treatment with the DNA-free™ DNase Kit (New England Biolabs, United States) to eliminate genomic DNA contamination. RNA purity was verified by measuring A260/A280 ratios (2.08–2.12) using a NanoDrop™ UV spectrophotometer (Thermo Fisher Scientific, United States). To confirm the absence of residual DNA, RNA samples not subjected to reverse transcription were amplified by PCR using gene-specific primers employed in this study.

First-strand cDNA was synthesized from 1 μg of purified RNA using the THERMOscript™ 1st Strand cDNA Synthesis Kit (Aidlab Biotechnologies, China) following the manufacturer’s protocol. Quantitative PCR (qPCR) was performed on a 7500 FAST Real-Time PCR System (Applied Biosystems, United States) with primers targeting denitrification-related genes, phenazine biosynthesis genes, quorum sensing genes and the reference genes *proC* (see Supplementary material). The qPCR conditions and cycling parameters were implemented as described by Nie et al. (2021), with cDNA serving as the template for amplification.

### 2.5 Untargeted metabolomics analysis

#### 2.5.1 Samples treatment

For metabolomic extraction, 50 mg of cell pellets were weighed into a 2 mL centrifuge tube containing a 6-mm-diameter grinding bead. A 400 μL extraction solution (methanol:water = 4:1, v/v) spiked with 0.02 mg/mL internal standard (L-2-chlorophenylalanine) was added to the tube. The sample was homogenized in a frozen tissue grinder for 6 min at −10°C and 50 Hz, followed by low-temperature ultrasonic extraction for 30 min at 5°C and 40 kHz. The mixture was then incubated at −20°C for 30 min and centrifuged at 13,000 × g for 15 min (4°C). The supernatant was transferred to an autosampler vial with an insert for subsequent instrumental analysis. A quality control (QC) sample was prepared by mixing equal volumes of metabolites from all samples. During instrumental analysis, a QC sample was inserted every 3 samples to evaluate the reproducibility of the entire analytical process.

#### 2.5.2 Liquid chromatography-tandem mass spectrometry analysis

The LC-MS/MS analysis of the samples was performed using a Thermo Fisher Scientific UHPLC-Q Exactive HF-X system (Shanghai Majorbio Bio-pharm Technology Co., Ltd.). The samples were separated on an HSS T3 chromatographic column (100 mm × 2.1 mm i.d., 1.8 μm) with a mobile phase A consisting of 95% water + 5% acetonitrile (containing 0.1% formic acid) and mobile phase B consisting of 47.5% acetonitrile + 47.5% isopropanol + 5% water (containing 0.1% formic acid). The flow rate was 0.40 mL/min, and the column temperature was maintained at 40°C.

Mass spectrometry conditions: Data acquisition was performed in both positive and negative ion scanning modes with a mass scan range of 70–1,050 m/z. The sheath gas flow rate was set at 50 psi, auxiliary gas flow rate at 13 psi, and auxiliary gas heater temperature at 425°C. The ion spray voltage was set to 3,500 V in positive mode and −3500 V in negative mode. The ion transfer tube temperature was maintained at 325°C, and a stepped collision energy of 20–40–0 V was applied. The full scan mass spectra were acquired at a resolution of 60,000 (MS1) and 7,500 (MS2), respectively. Data-dependent acquisition (DDA) mode was employed for data collection.

#### 2.5.3 Material identification and analysis

The LC-MS raw data were imported into the metabolomics processing software Progenesis QI (Waters Corporation, Milford, United States) for baseline filtering, peak identification, integration, retention time correction, and peak alignment. This process ultimately generated a data matrix containing retention time, mass-to-charge ratio (m/z), and peak intensity. The MS and MS/MS spectra were matched against public metabolite databases (HMDB; Metlin)^1^ and Majorbio’s in-house library to obtain metabolite annotations.

The annotated data matrix was then uploaded to the Majorbio Cloud Platform (https://cloud.majorbio.com/page/flow/index.html) for analysis. Data preprocessing included: (1) Applying the 80% rule to remove missing values, retaining variables with non-zero values in at least 80% of samples within any experimental group; (2) Filling remaining missing values with the minimum value from the original matrix; (3) Performing sum normalization to minimize technical variations from sample preparation and instrument instability, resulting in a log10-transformed normalized matrix; (4) Removing variables with relative standard deviation (RSD) > 30% in quality control (QC) samples; (5) Final log transformation to generate the processed matrix for downstream analysis.

Statistical analysis was performed using the R package ropls (Version 1.6.2) to conduct Principal Component Analysis (PCA) and Orthogonal Partial Least Squares Discriminant Analysis (OPLS-DA). Model robustness was evaluated through 7-fold cross-validation. Significantly differential metabolites were identified based on variable importance in projection (VIP) scores from OPLS-DA and Student’s *t*-test *p*-values, with thresholds set at VIP > 1 and *p* < 0.05.

Pathway annotation of differential metabolites was performed using the KEGG database^2^. Pathway enrichment analysis was conducted via the Python scipy.stats package, with statistically relevant biological pathways identified through Fisher’s exact test to reveal treatment-related metabolic mechanisms.

## 3 Results and discussion

### 3.1 Phenazine synthesis and quorum sensing in MEC reactors with/without 0.8 V applied voltage

**FIGURE 1:**
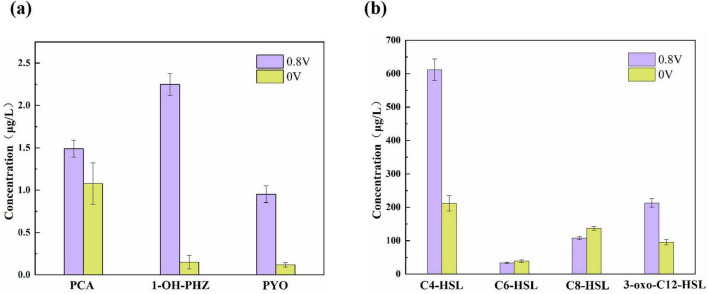
**(a)** Phenazine derivatives and **(b)** quorum sensing signal molecules detected in *Pseudomonas aeruginosa*-inoculated MEC reactors on day 72.

Phenazine derivatives and QS signal molecules were detected in *Pseudomonas aeruginosa*-inoculated MEC reactors on day 72 (Figure 1). In the MEC reactors with an applied voltage of 0.8 V, the production levels of PCA, 1-OH-PHZ, and PYO were 1. 38-, 14. 98-, and 8.65-fold higher, respectively, compared to those in the 0 V control reactors. The differences in QS signal molecules were primarily attributed to C4-HSL and 3-oxo-C12-HSL, which are secreted by *P. aeruginosa*, with increases of 2.88- and 2.21-fold, respectively. Additionally, trace amounts of other AHLs, including C6-HSL and C8-HSL, were detected at concentrations ranging from 34.23 to 39.41 μg/L and 12.14–23.15 μg/L, respectively. These AHLs may originate from other bacterial species within the microbial community.

**FIGURE 2:**
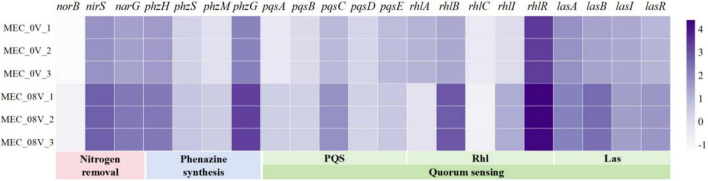
Relative expression of denitrification, phenazine synthesis, and quorum-sensing genes in *Pseudomonas aeruginosa* with/without 0.8 V applied voltage. Relative expression abundance processed with log10.

In the phenazine biosynthesis pathway of *P. aeruginosa*, the *phzG* gene is located upstream of all phenazine derivative synthesis pathways and participates in the production of the core intermediate PCA. Under an external voltage of 0.8 V, the expression level of *phzG* was upregulated by 14.8-fold (Figure 2); however, the production of PCA did not show a significant increase, likely because PCA, as an intermediate, is further metabolized by downstream regulatory genes. The genes *phzH*, *phzS*, and *phzM* regulate the conversion of PCA into PCN, PYO, and 1-OH-PHZ, respectively. Their expression levels were upregulated by 5. 38-, 2. 02-, and 3.69-fold, respectively (Figure 2). These expression changes exhibited a certain correlation with the altered production levels of their corresponding phenazine derivatives.

In addition, genes related to QS all showed varying degrees of upregulation, including the PQS, Las, and Rhl systems (Figure 2). Among these, the genes in the Rhl system exhibited the most significant upregulation trend: the genes regulating C4-HSL synthesis, rhlB and rhlI, were upregulated by 36.29- and 15.2-fold, respectively, while the gene regulating C4-HSL-binding protein synthesis, rhlR, was upregulated by 9.15-fold. The Las system holds the highest priority among these three QS systems and can positively regulate the Rhl and PQS systems through feedback mechanisms (Miller and Bassler, 2001; Soukarieh et al., 2018). In contrast, the Rhl system exerts negative feedback regulation on the PQS system (Diggle et al., 2006). Under applied voltage, this negative feedback regulation may be attenuated due to the combined effects of Las-mediated positive feedback regulation and intrinsic enhancement of the PQS system.

### 3.2 Differential metabolites analysis in MEC reactors with/without 0.8 V applied voltage

**FIGURE 3:**
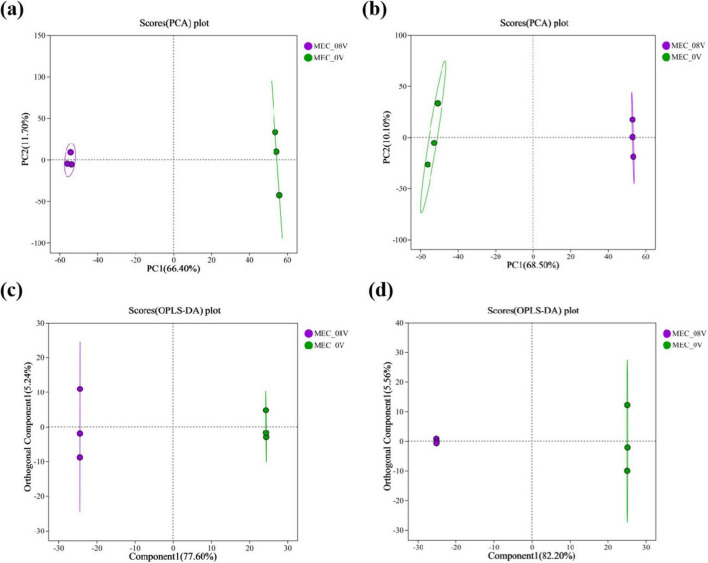
Score plot of principal component analysis (PCA) for metabolites in MEC reactors with/without 0.8 V applied voltage **(a)** positive ion mode **(b)** negative ion mode; score plot of orthogonal partial least squares discriminant analysis (OPLS-DA) for metabolites in MEC reactors with/without 0.8 V applied voltage **(c)** positive ion mode **(d)** negative ion mode.

**FIGURE 4:**
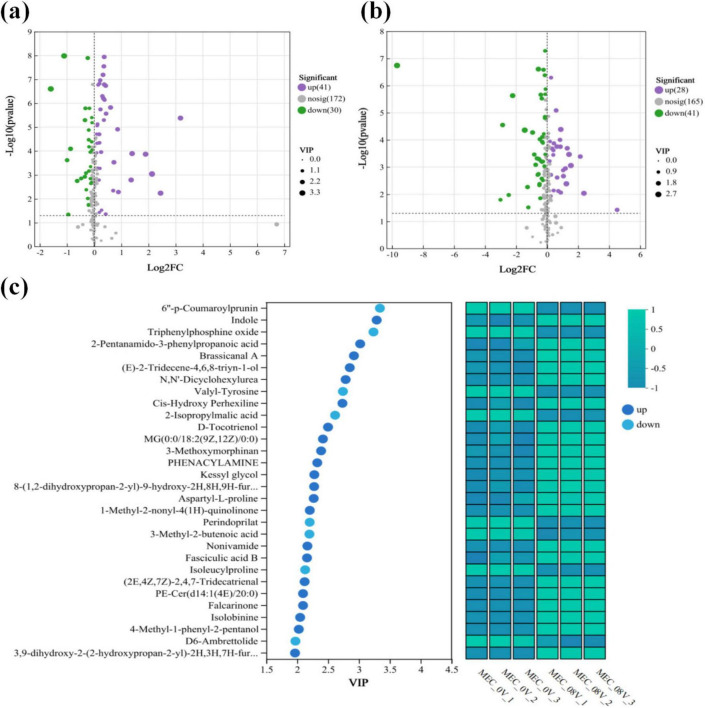
Volcano plots of differential metabolites in **(a)** positive ion mode; **(b)** negative ion mode. The x-axis represents the log2-transformed fold change (log2FC) of metabolite expression between groups, and the y-axis corresponds to the statistical significance of expression changes [-log10 (*p*-value)]. Each point denotes a specific metabolite, with size indicating VIP scores. Purple, green, and gray dots represent significantly upregulated, downregulated, and non-significant differential metabolites, respectively. **(c)** Bubble plot and relative expression heatmap of the top 30 VIP-ranked significant differential metabolites.

Principal Component Analysis (PCA) and Orthogonal Partial Least Squares-Discriminant Analysis (OPLS-DA) were performed on the metabolomic profiles of MEC reactors under 0.8V applied voltage and non-electrified conditions. Notably, in both positive and negative ion modes, the electrified group exhibited clear separation from the control group along the first principal component (PC1), demonstrating that voltage application significantly altered the metabolic landscape (Figure 3). Among the 477 annotated metabolites detected, 69 differential metabolites were significantly upregulated (41 in positive ion mode and 28 in negative ion mode), and 71 were significantly downregulated (30 in positive ion mode and 41 in negative ion mode), as illustrated in the volcano plots (Figures 4a,b).

Among the top 30 differential metabolites ranked by VIP value, Indole is the top-ranked upregulated metabolite (Figure 4c). Indole and its derivatives represent a class of microbial signaling molecules, with a notable example being indole-3-acetic acid (IAA), a classic plant growth hormone. Recent studies have also linked indole to quorum sensing, and its addition during water treatment has been shown to enhance biofilm growth in *Burkholderia* (Wu et al., 2024). Indole can also directly stimulate biofilm formation in *P. aeruginosa* and *Streptococcus mutans* (Inaba et al., 2020; Kim et al., 2015). Additionally, another upregulated metabolite identified among the top 30 VIP-ranked differential metabolites is brassicanal A, a metabolic derivative of indole that is closely associated with indole biosynthesis (Wang et al., 2023). However, the majority of other differential metabolites have only been identified in applications such as material synthesis and pharmaceutical synthesis, with no clear link established to microbial activities, particularly quorum sensing. Therefore, further analysis of the functional roles of differential metabolites with VIP values beyond the top 30 remains necessary.

**FIGURE 5:**
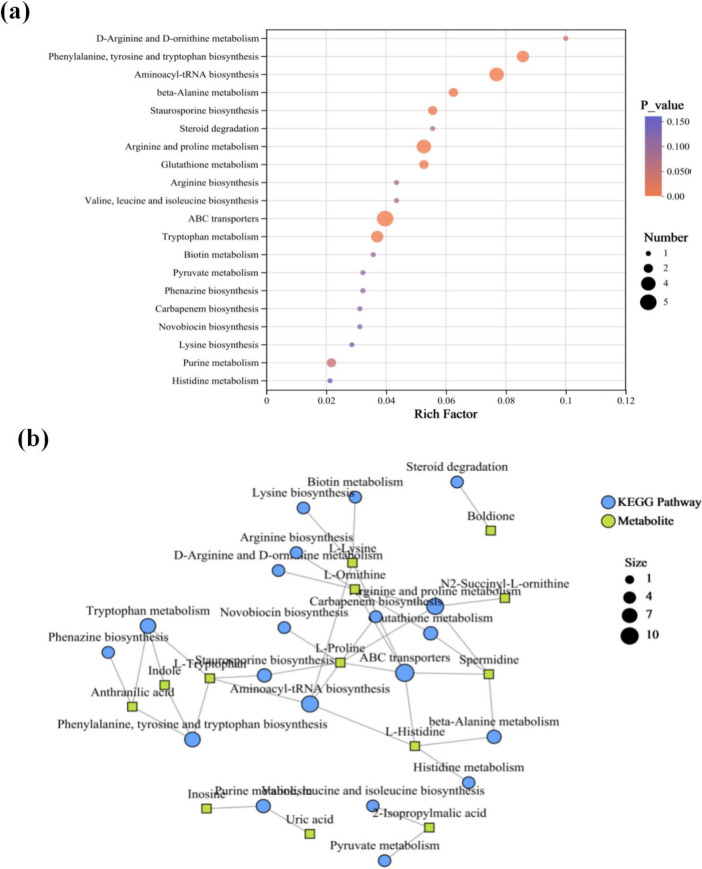
**(a)** KEGG enrichment bubble plot of differential metabolites. X-axis: Enrichment ratio (calculated as number in study group/number in population); Y-axis: KEGG pathways. Bubble size represents the number of metabolites enriched in each pathway; color indicates the enrichment significance [-log10 (*p*-value)]. **(b)** KEGG enrichment network diagram. Green square nodes denote metabolites; blue circular nodes represent KEGG pathways, with node size proportional to the number of associated metabolites.

Thirteen differential metabolites, including indole, L-ornithine, anthranilic acid, L-tryptophan, L-histidine, L-proline, spermidine, boldione, N2-succinyl-L-ornithine, 2-isopropylmalic acid, uric acid, inosine, and L-lysine, were annotated with KEGG among the 140 differential metabolites, and these exhibited enrichment across 18 metabolic pathways (Figure 5a). Nearly half of the differential metabolites were amino acids and their derivatives. Consequently, pathways related to amino acid synthesis and transport—including D-arginine and D-ornithine metabolism, phenylalanine, tyrosine and tryptophan biosynthesis, aminoacyl-tRNA biosynthesis, beta-Alanine metabolism, arginine and proline metabolism, and ABC transporters—exhibited high enrichment factors. Under electrical stimulation, granular sludge or biofilms show increased secretion of extracellular polymeric substances (EPS), whose primary components are polysaccharides (PS) and proteins (PN) (Jiang et al., 2023; Liu et al., 2019). These components mediate material exchange between bacterial cells and the external environment, which may explain the observed hyperactivity in amino acid synthesis and transport metabolism. Another noteworthy observation is the pathways associated with indole, specifically phenylalanine, tyrosine and tryptophan biosynthesis, and tryptophan metabolism, both of which are linked to L-tryptophan and anthranilic acid (Figure 5b). Anthranilic acid is associated with phenazine biosynthesis. Its precursor, chorismate, can simultaneously participate in the synthesis of phenazine derivatives and the conversion to anthranilic acid, ultimately generating the PQS signaling molecule. Under electrical stimulation, the upregulation of anthranilic acid may coincide with enhanced activation of the PQS system. Additionally, L-tryptophan, as a downregulated differential metabolite in this study, can be degraded by certain bacteria to produce anthranilic acid and indole. The observed reduction in its accumulation might result from intensified degradation processes.

### 3.3 Correlation analysis between differential metabolites and microbial communities in MEC reactors

**FIGURE 6:**
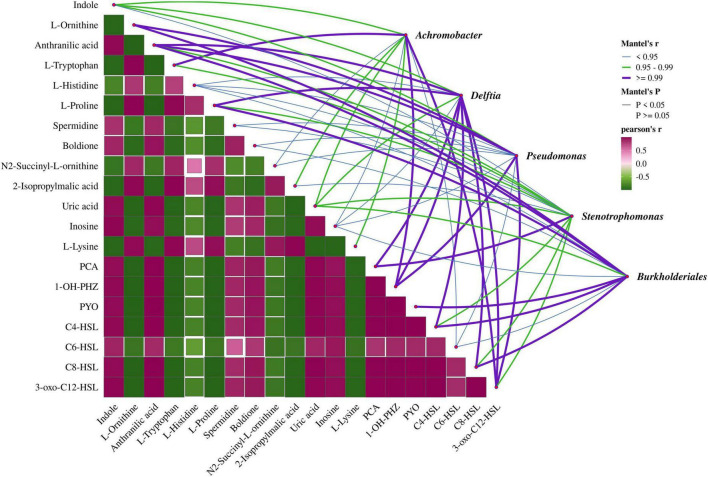
Correlation analysis of differential metabolites with microbial communities in MEC reactors. Analysis based on Pearson correlation coefficients; Mantel tests for correlations between metabolites and microbial communities. The line width and color represent the Mantel’s r statistic for the corresponding microbial community and environmental factor, while the line style indicates the significance of the statistical analysis.

To clarify the association between differential metabolites and microbial communities in MEC reactors, a Mantel test-based correlation analysis was conducted (Figure 6). After 72 days of operation, four bacterial genera, *Achromobacter*, *Delftia*, *Stenotrophomonas*, and *Burkholderiales*, were identified in MEC reactors inoculated with *Pseudomonas aeruginosa* PAO1. Among these, the abundances of *Pseudomonas*, *Delftia*, and *Burkholderiales* were higher in reactors with an applied voltage of 0.8 V. The analysis revealed that the metabolites most strongly correlated with these genera were primarily QS-related compounds, such as anthranilic acid, phenazine derivatives, and QS signaling molecules. Additionally, amino acids involved in metabolic processes, including L-ornithine, L-tryptophan, and L-proline, exhibited significant correlations.

The three genera whose abundances were enhanced by electrical stimulation, *Pseudomonas*, *Delftia*, and *Burkholderiales*, exhibited strong correlations with multiple metabolites. Delftia is recognized as a genus with quorum quenching (QQ) activity, enabling it to degrade AHLs and compete with QS bacteria like *Pseudomonas aeruginosa* in most environments (Singh et al., 2017; Xu et al., 2023). However, under electrical stimulation, *P. aeruginosa* released excessive AHLs, and the relatively low abundance of *Delftia* (7.14%) was insufficient to fully degrade these AHLs. This resulted in a transient synergistic relationship between *P. aeruginosa* and *Delftia*, leading to a close correlation between Delftia’s abundance and the levels of C4-HSL and 3-OXO-C12-HSL secreted by *P. aeruginosa*. In contrast, *Burkholderiales* was hypothesized to exhibit a QS-like system mediated by indole as a signaling molecule (Chunxiao et al., 2023; Wu et al., 2024). However, the correlation between indole and *Burkholderiales* was the weakest among all tested relationships. This may be attributed to the unclear biosynthetic origin of indole within the community structure of this study. In fact, indole synthesis is widespread among microbial genera; for example, *Escherichia coli* can synthesize indole via tryptophanase. However, within *Burkholderiales*, only a single *Burkholderia sp.* has been reported to produce indole (Lee and Lee, 2010), suggesting that it is unlikely derived from *Burkholderiales* itself.

The other two genera, *Achromobacter* and *Stenotrophomonas*, showed higher abundances under non-electrostimulated conditions. *Achromobacter* exhibited the strongest negative correlation with 3-OXO-C12-HSL. As a heterotrophic nitrifier and aerobic denitrifier, *Achromobacter* has been reported to enhance its growth activity and denitrification capacity when co-cultured with strains secreting C8-HSL (Zhao et al., 2022). However, since C8-HSL levels in the system were extremely low, *Achromobacter*’s dominance (46.4% abundance without electrical stimulation) may stem from its inherent competitive advantage over *P. aeruginosa*. Under electrical stimulation, *P. aeruginosa*’s growth was upregulated by 3-OXO-C12-HSL, suppressing *Achromobacter*’s abundance to 12.9%. Additionally, certain strains of the genus *Achromobacter*, such as *Achromobacter* sp. strain N2, have been reported to harbor metabolic pathways for synthesizing IAA and are capable of forming symbiotic relationships with plants (Corsini et al., 2018). Therefore, the indole detected in the system may originate from direct electrochemical conversion. *Stenotrophomonas* displayed the weakest correlation with differential metabolites among the five genera. This is likely because *Stenotrophomonas* primarily employs the diffusible signal factor (DSF) system for QS, and its ecological niche depends on the cooperative and competitive dynamics of the other four genera (Coves et al., 2023; Zhuang et al., 2023).

## 4 Conclusion

This study demonstrates that applying 0.8 V voltage significantly enhances phenazine synthesis and quorum sensing (QS) activity in *Pseudomonas aeruginosa* PAO1 within MEC reactors. Key phenazine derivatives (e.g., PYO, 1-OH-PHZ) and QS signaling molecules (C4-HSL, 3-OXO-C12-HSL) increased by 8.65–14.98-fold and 2.21–2.88-fold, respectively, accompanied by upregulation of biosynthesis genes (*phzG*, *rhlI*). Electrical stimulation amplified QS network interactions by reinforcing Las-mediated positive feedback on Rhl/PQS systems while weakening Rhl’s inhibitory effect on PQS. Metabolomics revealed differential metabolites (e.g., indole, anthranilic acid) enriched in amino acid and indole-related pathways, linked to upregulated extracellular polymeric substance (EPS) secretion and substrate competition under voltage. Microbial community analysis indicated voltage-driven enrichment of *Pseudomonas*, *Delftia*, and *Burkholderiales*. *Delftia* formed transient synergism with PAO1 due to incomplete AHL degradation, while *Burkholderiales* showed weak indole correlation, likely due to non-indigenous origin. In contrast, *Achromobacter* dominance (46.4% without voltage) was suppressed (12.9% with voltage) by PAO1’s 3-OXO-C12-HSL-mediated growth advantage. These findings highlight that voltage reshapes metabolic networks and microbial interactions, optimizing functional community structure and metabolic efficiency in MEC reactors, providing insights for electro-stimulated biofilm engineering.

## Data Availability

The original contributions presented in this study are included in this article/Supplementary material, further inquiries can be directed to the corresponding author.

## Footnotes

1
https://metlin.scripps.edu/

2
https://www.kegg.jp/kegg/pathway.html
