# Supplementary material for: Integrated analysis of electrical stimulation effects on Pseudomonas aeruginosa PAO1 inoculated denitrifying community: targeted and untargeted metabolomic analysis of phenazine biosynthesis and quorum sensing
Source: Front Microbiol. 2025 Jun 13;16:1621417. doi: 10.3389/fmicb.2025.1621417 (PMC12202436; doi:10.3389/fmicb.2025.1621417)
Supplement: Supplementary file 1 [file Supplementary_file_1.docx]

**Integrated analysis of electrical stimulation effects on *Pseudomonas aeruginosa* PAO1 inoculated denitrifying community: targeted and untargeted metabolomic analysis of phenazine biosynthesis and quorum sensing**

Li Wu^a,b^, Yong Liu^a,b^, Jianping Deng^a,b^, Shuanglin Gui^a,b*^, Hanbing Nie^a,b*^

^a^ Jiangxi Provincial Key Laboratory of Greenhouse Gas Accounting and Carbon Reduction, Institute of Energy Research, Jiangxi Academy of Sciences, Nanchang 330096, China

^b^ Jiangxi Carbon Neutalization Research Center, Nanchang 330096, China

*Corresponding authors

Hanbing Nie, Institute of Energy Research, Jiangxi Academy of Sciences, 7777 Changdong Avenue, Nanchang 330096, China (E-mail: [niehanbing221@126.com)](mailto:niehanbing221@126.com))

**Appendix A:**

***Supplementary Tables***

**Table S1.** Primer sequences used in this study

| qRT-PCR of housekeeping genes | Primer sequence |
| --- | --- |
| proC_649f | CTGTCCAGCGAGGTCGAG |
| proC_822r | TTATTGGCCAAGCTGTTCG |
| qRT-PCR of denitrification gene |  |
| *narG*_1828f | AACGGCACCAGCTTCTTCTA |
| *narG*_1950r | GTTGTAGTCCAGGGCGTGTT |
| *nirS*_1293f | GCAGTACGCCTGGAAGAAAG |
| *nirS*_1395r | GGTGGTGTCGACGTAGAGGT |
| norB_567f | CTACAACCCGGAAAACCTCA |
| norB_688r | TGATCTTCACCAGCACGAAG |
| qRT-PCR of phenazine synthesis genes |  |
| phzG_528f | GTTCGAACTGTGCCTGGAGT |
| phzG_640r | GTTGCAGGTAGCGGTGCT |
| phzM_206f | AGATCTTCCAGGGCGATACC |
| phzM_307r | CGTAGAACAGCACCATGTCG |
| phzS_9F | ACCCATCGATATCCTCATCG |
| phzS_121R | GTATCTCGCTGCTGCTTTCC |
| phzH_1478f | ATGAGCTGGTGGAGTACGTC |
| phzH_1624r | TGGCAGAAGTCGGATAAGGG |
| qRT-PCR of quorum sensing genes |  |
| lasA_178F | GACGACCTGTTCCTCTACGG |
| lasA_280R | GCTCCAGGTATTCGCTCTTG |
| lasB_1036F | CAGAACTCCGGGCTGATCTA |
| lasB_1170R | GCCCTTCTTGATGTCGTAGC |
| lasI_94F | GGCTGGGACGTTAGTGTCAT |
| lasI_197R | AAAACCTGGGCTTCAGGAGT |
| lasR_33F | ACGCTCAAGTGGAAAATTGG |
| lasR_143R | TCGTAGTCCTGGCTGTCCTT |
| rhlA_661F | AGCTGGGACGAATACACCAC |
| rhlA_770R | GACTCCAGGTCGAGGAAATG |
| rhlB_339F | CATCGCTCACGAGAAGTACG |
| rhlB_438R | GTTGAACTTGGGGTGTACCG |
| rhlC_422F | TCAGGCAATTGTCTCTGGAC |
| rhlC_566R | GTCCACGTGGTCGATGAAC |
| rhlI_13F | CTCTCTGAATCGCTGGAAGG |
| rhlI_157R | GATGGTCGAACTGGTCGAAT |
| rhlR_112F | CTGGGCTTCGATTACTACGC |
| rhlR_234R | CCCGTAGTTCTGCATCTGGT |
| pqsA_384F | CCTTTGCTCGACGATTTCTC |
| pqsA_502R | GTGGAACCCGAGGTGTATTG |
| pqsB_283F | GTCTTCGACCTCACCGACTC |
| pqsB_415R | ACTCGCTGTCCACTTCCAAT |
| pqsC_366F | ATTGGATTCGCAGATGGAGT |
| pqsC_480R | GGAGATGTACTCGCTGCACA |
| pqsD_519F | GAGTCTCGAAGACGGACTGC |
| pqsD_631R | CATTCTCGTCGAGGAAGGTC |
| pqsE_106F | CTGGTTGAAGGAGGGATCAG |
| pqsE_219R | GTCGTAGTGCTTGTGGGTGA |
